# Malariometric Indices among Nigerian Children in a Rural Setting

**DOI:** 10.1155/2013/716805

**Published:** 2013-02-28

**Authors:** Ekong E. Udoh, Angela E. Oyo-ita, Friday A. Odey, Komomo I. Eyong, Chioma M. Oringanje, Olabisi A. Oduwole, Joseph U. Okebe, Ekpereonne B. Esu, Martin M. Meremikwu, Asindi A. Asindi

**Affiliations:** ^1^Department of Paediatrics, University of Calabar Teaching Hospital, PMB 1278, Calabar, Nigeria; ^2^Calabar Institute of Tropical Disease Research and Prevention, University of Calabar Teaching Hospital, P.O. Box 1211, Calabar, Nigeria; ^3^Department of Community Medicine, University of Calabar Teaching Hospital, PMB 1278, Calabar, Nigeria; ^4^Medical Research Council (UK), Fajara, P.O. Box 273, Banjul, Gambia

## Abstract

Malaria contributes to high childhood morbidity and mortality in Nigeria. To determine its endemicity in a rural farming community in the south-south of Nigeria, the following malariometric indices, namely, malaria parasitaemia, spleen rates, and anaemia were evaluated in children aged 2–10 years. This was a descriptive cross-sectional survey among school-age children residing in a rubber plantation settlement. The children were selected from six primary schools using a multistaged stratified cluster sampling technique. They were all examined for pallor, enlarged spleen, or liver among other clinical parameters and had blood films for malaria parasites. Of the 461 children recruited, 329 (71.4%) had malaria parasites. The prevalence of malaria parasitaemia was slightly higher in the under fives than that of those ≥5 years, 76.2% and 70.3%, respectively. Splenic enlargement was present in 133 children (28.9%). The overall prevalence of anaemia was 35.7%. Anaemia was more common in the under-fives (48.8%) than in those ≥5 years (32.8%). The odds of anaemia in the under fives were significantly higher than the odds of those ≥5 years (OR = 1.95 [1.19–3.18]). Malaria is highly endemic in this farming community and calls for intensification of control interventions in the area with special attention to school-age children.

## 1. Introduction

Malaria remains a leading cause of illness and death in sub-Saharan Africa with the greatest risk seen in children under the age of five, pregnant women, and people living with HIV/AIDS [1, 2]. About 50% of Nigerians are estimated to have at least one episode of the disease each year with over 200,000 deaths in children annually [3]. Estimates of malaria burden are based on malariometric indices like prevalence of malaria parasitaemia, spleen rate, and anaemia in defined risk groups [2]. School-age children are vulnerable to the disease and have been studied over the years to determine malaria burden at community levels using these malariometric indices [4]. 

An understanding of the malaria burden in a given setting is important for health planning, policy development, and control interventions. This study aimed at determining the malaria burden at Ikot-Omin, a rural rubber plantation settlement, using malaria parasitaemia, spleen rate and anaemia as malariometric indices.

## 2. Methods

As part of site preparation for the setting up of a sentinel site for monitoring antimalarial efficacy and drug resistance [5], we conducted a cross-sectional study in Ikot-Omin; a suburb of Calabar in Cross River state, Southern Nigeria. The area is a rubber plantation settlement located 20 kilometres from Calabar, the capital city. It is located at the tropical rain forest belt with average temperature and relative humidity of 28.0°C and 80.5%, respectively. The main malaria vectors in the area are *Anopheles gambiae* and *Anopheles funestus.* Parasite resistance to chloroquine and sulfadoxine/pyrimethamine was over 80% [6, 7]. The survey was conducted in January 2008.

Children aged 2–10 years attending primary schools in the area were considered eligible for inclusion in the study. They were selected using a multistage stratified cluster sampling technique. The sample size in the study was calculated based on prevalence of malaria parasitaemia in a previous study in a similar setting [8]. Ethical clearance was obtained from the Health Research Ethics Committee of the University of Calabar Teaching Hospital. Children were recruited into the study after parental informed consent had been obtained. A general examination was done, and anthropometric measurements: weight, height, mid-arm circumference (for children between 2 and 5 years), and temperature were taken. Assessment for spleen enlargement was done in all the children following standard methods [9]. Thick and thin blood films were prepared for malaria microscopy and stained using Giemsa. Two microscopists independently read each slide for parasite until 200 white blood cells (WBC) were counted. Discrepancies in the parasite count were resolved by a third microscopist. Parasite density was determined by dividing the parasite count by 200 and multiplying by 8,000 (approximate number of WBCs/microliter) [10]. Blood was also sampled for packed cell volume (PCV). Children with malaria parasitaemia were treated with an appropriate dose of either artemether/lumefantrine or artesunate/amodiaquine combination based on the country's national treatment policy for uncomplicated malaria [11].

The data was double entered and analyzed using Epi-Info version 3.3.2 [12]. The prevalence of malaria parasitaemia, spleen rate, and anaemia (PCV < 30%) was calculated as a proportion of children with those indices. Chi square was used for association between categorical variables, while student's *t*-test was used in the test of significance for the difference between two means.

## 3. Results

A total of 461 children participated in the study. The PCV of 18 of them (3.9%) could not be obtained either due to breakage of capillary tubes or spillage of blood sample during centrifugation. These were excluded from analysis related to anaemia and mean haematocrit estimation. Two hundred and twenty-nine were males (49.7%). Eighty-four (18.2%) were under fives, while the rest were aged 5–10 years. The mean age of the study population was 6.48 ± 2.1 years. 

A positive film was reported in 329 (71.4%). All had *P. falciparum *monoinfection. The prevalence of parasitaemia was slightly higher in the under fives (76.2%) compared with those of 5–10 years of age (70.3%). The spleen rate was 28.9%. There was no significant difference in spleen rate between the under fives (31.0%) and those of 5–10 years of age (28.4%). The prevalence of anaemia was 48.8% in the under fives, 32.8% in those of 5−10 years of age, and 35.7% for both age categories. The odds of malaria parasitaemia or splenomegaly between the under-fives and those of five years and above were not significantly different. However, the odds of anaemia in the under fives was significantly higher than those of five years and above (OR = 1.95 [1.19–3.18]) (Table 1).

There was no significant difference in the mean haematocrit value between the parasitaemic and aparasitaemic children (*t* test = 0.02; *P* value = 0.98) as shown in Table 2.

## 4. Discussion

The overall prevalence of malaria parasitaemia in the study population was 71.4%. The prevalence of malaria parasitaemia was slightly higher in the under fives compared with those aged 5–10 years. This is consistent with the general observation that the under fives are more vulnerable to the disease in areas of high transmission [13, 14]. Naturally acquired immunity builds up in older children following repeated exposure to the parasite and is manifested by lower parasite densities and fewer clinical malarial episodes than younger children and less exposed under fives [15].

The prevalence of malaria in this study is much higher than the 59.6% obtained about a decade ago in a community close to where this study was done [8]. Our finding is also higher than 48.3% recorded by Wang et al. [16] in a multicenter study conducted among school children aged 6−10 years living in some East and West African countries. *P. falciparum* was the only species of malaria parasite identified in this study against *P. falciparum* and *P. malariae* reported in a previous study in a nearby community [8]. 

The high prevalence of malaria and *P. falciparum *monoinfection noted in this study is an indication of a worsening malaria situation around the study area which might be indicative of poor access or adherence to effective control measures such as the use of insecticide treated nets (ITNs). The results of a more recent study, National Malaria Indicator Survey (MIS), have shown an improvement in this situation with a national average prevalence of malaria parasitaemia in preschool children of 42% [17]. The result of the MIS, however, showed that the prevalence of malaria parasitaemia was the highest (50%) among children from the lowest wealth quintile. The high prevalence reported in our study population could also be partly explained by the fact that the study population was predominantly poor and rural. 

 The overall spleen rate in this study is 28.9%. The under fives had a marginally higher spleen rate (31%) compared with those of 5−10 years of age (28.4%). These findings buttress the fact that under fives have a more pronounced immunological response (including splenic enlargement) to malaria than the older children [14]. The spleen rate in our study is much higher than the 11.7% obtained by Adeyemo et al. [18] among apparently healthy children, from a rural primary school in south west Nigeria but less than 44% and 74.5% reported in Sierra Leone and India, respectively [19, 20]. The spleen rate is usually high in areas of high malaria transmission and antimalarial treatment failure. Persistent parasitaemia from treatment failure is known to contribute to splenic enlargement, and this was the case in the India study site [20]. With the introduction of ACTs as the first-line treatment for malaria in Nigeria the spleen rate in under-five children is expected to further decline as a prompt access to effective treatment improves. 

Anaemia occurred in 35.7% of the study population with the under fives having a higher prevalence (48.8%) compared with children of five years and above (32.8%). The under fives were about twice more likely to be anaemic compared with those of five years and above. These observations agree with the fact that this age group is more vulnerable to *P. falciparum* infection than others with severe and potentially fatal complications ensuing [14]. The prevalence of anaemia among under fives in our study was much lower than 72% obtained in the national MIS [17]. The difference in the definition and method of determining anaemia between the National MIS and our study is partly responsible for the marked disparity in the results. Whereas the National MIS used a HemoCue to estimate haemoglobin concentration and defined anaemia as Hb < 11.0 g/dL in under fives or Hb < 11.5 g/dL in those of 5–11 years, we used a microematocrit centrifuge to estimate the PCV of the children and defined anaemia as PCV < 30%. The prevalence of anaemia in under fives in this study was comparable with similar studies conducted in Ghana [21]. Studies conducted in India and Venezuela recorded higher prevalence of anaemia in children than what was found in our study [22, 23]. Dietary inadequacy involving all nutrients including iron, high intake of tea which tends to inhibit iron absorption, and poverty were the main reasons for anaemia among children in the Indian study, while in the Venezuela, malaria was reported as the main cause of anaemia [22, 23]. 

This study shows a marked disparity in the malariometric indices assessed in the children. The highest being the prevalence of malaria parasitaemia (71.4%), followed by anaemia (35%), and then spleen rate (28.9%). The prevalence of malaria parasitaemia may have been influenced by antimalarial drug use in the community, anaemia by helminthic infestation and dietary intake and spleen rate by the extent and duration of exposure to the parasite [24, 25]. Malaria would have been taken to be mesoendemic in our study area if endemicity was assessed by only the spleen rate and anaemia. Such a conclusion would have misinformed policy makers when deciding on the control measures to implement in that area. Since there is variability in the sensitivity of these indices, multiple estimates of endemicity should be assessed, and the parameter with the highest prevalence be used to inform decision on interventions. This study was conducted in January which is a relatively low malaria transmission period in the country [26]. Since there is seasonal variability in malaria transmission in the country, reporting on point prevalence of malaria parasitaemia is acknowledged as a limitation of the study in determining the endemicity of the disease.

There was no statistically significant difference in the mean haematocrit value between parasitaemic and aparasitaemic children in this study, though some studies have reported such a difference between them [14, 23]. The inability to detect a difference in the mean haematocrit value between both categories of children in this study could be due to the low-grade parasitaemia among those with malaria or that factors other than malaria which were not elucidated in this study might have influenced our finding.

## 5. Conclusions

This study shows that malaria remains a major health problem among school-age children residing in agricultural settlements in the south-south of Nigeria. It is therefore necessary to intensify malaria control efforts with special attention given to school-age children residing in at-risk environments. 

Further studies on aetiology of anaemia in children using a uniform methodology are necessary for the development of effective preventive and therapeutic interventions for anaemia in school-age children residing in such environments.

## Figures and Tables

**Table 1:** Prevalence of malariometric indices in children.

Age (years)	Malaria parasitaemia^a^			Splenomegaly^b^			Anaemia^c^		
	Present %	Absent	OR [95% CI]	Present %	Absent	OR [95% CI]	Present %	Absent	OR [95% CI]
<5	64 (76.4)	20	1.35 [0.78–2.34]	26 (31.0)	58	1.13 [0.68–1.89]	39 (48.8)	41	1.95 [1.19–3.18]
≥5–10*	265 (70.3)	112		107 (28.4)	270		119 (31.6)	244	

Both age groups	329 (71.4)	132		133 (28.9)	328		158 (35.7)	285	

^a^χ^2^ = 1.17; *P* = 0.279 (not statistically significant).

^b^ *χ* ^2^ = 0.22; *P* = 0.638 (not statistically significant).

^c^χ^2^ = 7.28; *P* = 0.007 (statistically significant).

*Baseline age group for calculation of OR [95% CI].

Prevalence of anaemia was based on 443 children.

**Table 2:** Comparison of mean haematocrit values of parasitaemic and aparasitaemic children.

Mean Hct value (%)	Mp + ve	Mp − ve	*t*-test	*P*-value
Age < 5 years	32.5 ± 4.6	30.7 ± 7.1	1.33	0.19
Age 5–10 years	34.5 ± 4.0	34.7 ± 5.2	0.40	0.69

Both age groups	34.09 ± 4.21	34.1 ± 5.7	0.02	0.98
